# Investigating intraocular pressure fluctuations using corneal visualization Scheimpflug technology tonometry (CST) with varied resting intervals

**DOI:** 10.3389/fmed.2026.1906060

**Published:** 2026-09-09

**Authors:** Xiaoqin Chen, Hongsu Wang, Xingyi Guo, Lihua Li, Kaixuan Zhang, Han Yu Zhang, Andrew K. C. Lam

**Affiliations:** 1 Tianjin Eye Hospital, Tianjin Key Lab of Ophthalmology and Visual Science, Tianjin, China; 2 Institute of Optometry and Vision Science, School of Medicine, Nankai University, Tianjin, China; 3 Tianjin Eye Hospital Optometric Center, Tianjin, China; 4 School of Medicine, Nankai University, Tianjin, China; 5 School of Optometry, Hong Kong Polytechnic University, Hong Kong SAR, China

**Keywords:** bIOP, corneal visualization scheimpflug technology (Corvis ST), glaucoma, IOP, myopia

## Abstract

**Background:**

To investigate the effects of different resting intervals on successive intraocular pressure (IOP) and biomechanically corrected IOP (bIOP) measurements from corneal visualization Scheimpflug technology tonometry (CST).

**Methods:**

This was a prospective repeated-measures observational study in young healthy myopes. Three successive IOP and bIOP measurements were acquired using the CST in both eyes of each participant, using four intervals between three successive measurements in the following order, 5 min (T5), 2 min (T2), 30 s (T30), and immediately after the other (Ti). T5-1 was the baseline measurement followed by T5-2 5 minlater, and T5-3 after another 5-min interval. A similar approach was applied to other intervals. A 5-min resting period was provided between successive sets of triplicate measurements. IOP and bIOP results were compared with four intervals and three measurements as the main effects.

**Results:**

A total of 88 myopic participants were recruited. Their mean age was 21.5 ± 2.5 years (from 19 to 31 years). There were 68 right eyes and 20 left eyes, with a spherical equivalent of −4.49 ± 2.39 D (from −9.88 to −0.50 D). A generalized linear mixed model showed that the order of interval did not show significant effect on IOP (*p* = 0.404), nor the measurement interval (*p* = 0.074). For bIOP, interval did not show significant effect (*p* = 0.325) whereas difference in measurement interval, and the effect of successive measurement approached statistical significance (*p* = 0.054). There was a decreasing trend of bIOP when measurement intervals were 2 min (Friedman test, *p* < 0.001) and 30 s (Friedman test, *p* = 0.035). The difference was less than 1 mmHg.

**Conclusion:**

Both IOP and bIOP obtained from CST were stable across successive measurement. Although bIOP decreased slightly with 2-min and 30-s, the reduction was small and not clinically significant.

## Introduction

Primary open-angle glaucoma (POAG) is a chronic, progressive optic neuropathy characterized by degeneration of the optic nerve rim and retinal nerve fiber layer, with/corresponding visual field defects (1). Glaucoma remains the most common cause of irreversible blindness worldwide (2, 3), and it has been projected that the global prevalence of glaucoma will rise to 76 million by 2020 and escalate to 111.8 million by 2040 (4). Intraocular pressure (IOP) is a key indicator of the equilibrium between the production and outflow of aqueous humour. Dysregulation of IOP can precipitate optic nerve damage and result in the visual field loss (2). Although retinal ganglion cell degeneration and vision loss may persist in patients even with well-controlled IOP (5), IOP reduction remains the only clinically proven approach to prevent glaucoma progression (6). Therefore, the accurate measurement of IOP is essential in the diagnosis and management of glaucoma.

The traditional method for IOP assessment, Goldmann applanation tonometry (GAT), has been well-established. However, repeated tonometry can reduce IOP, a phenomenon known as the “Bechrakis effect” (7–10). This effect has been attributed to an artificial drop of IOP during measurement, which leads to the expulsion of aqueous humour from the anterior chamber. The Bechrakis effect has also been observed in association with occulopression (11), the use of topical anesthetics, variations in central corneal thickness (CCT), and differences in the degree of fluorescein staining (12, 13).

With the advent of corneal visualization Scheimpflug technology tonometry (CST), a non-contact and less invasive approach to IOP measurement has become available (14). CST enables dynamic corneal deformation analysis under air puff stimulation, providing a rapid and repeatable assessment of IOP and corneal biomechanical parameters (14). Numerous studies examining CST repeatability have used variable intervals between repeated measurements, ranging from immediately consecutive measurements (15), a 1-min interval (16), a 3-min interval (17), or a 2- to 5-min interval (18). Lu et al. (19) did not report the interval between two IOP measurements.

While these protocols generally reported good reliability for IOP, the inconsistency in measurement intervals limits understanding of the effect of timing on repeatability. It has been documented that after a strong air pules, the cornea moves inwards until it reaches maximum deformation before returning to its original state (20). A previous study indicated that IOP could be reduced after a single CST measurement due to significant corneal deformation (21). However, using a 1-min interval between measurements yielded reliable IOP readings, with no significant changes observed among the three repeated measurements (22). In contrast, Borroni et al. (23) reported reductions in IOP, with a 21% decrease at 4 min and a 16% decrease at 8 min following CST measurements in healthy and glaucomatous middle-aged adults, respectively. Their post-CST IOP values obtained by rebound tonometry may be overestimated in highly myopic eyes (24, 25) and underestimated in low myopes (25). This discrepancy is mainly attributed to the influence of altered corneal biomechanics on rebound tonometry readings, rather than changes in CCT alone (26). Consequently, biomechanically corrected intraocular pressure (bIOP), which has been shown to be less influenced by CCT and corneal biomechanics (27–29), thereby providing a more accurate assessment of the true IOP.

There remains a need to systematically investigate how different intervals influence IOP measurement consistency. Therefore, the present study aimed to investigate the effect of different intervals between successive CST measurements on both IOP and bIOP. This investigation may contribute to a better understanding of the dynamics of IOP measurement and inform clinical guidelines for the use of CST in routine practice and research settings.

## Methods

It was a prospective repeated-measures observational study. Young healthy myopes aged between 18 and 35 years and with a spherical equivalent refractive error (SER) of at least −0.50D were recruited. Participants with prior ocular surgery, ocular disease, or contact lens wear within the past three months were excluded. Refractive error was measured with an open-field autorefractor (Shin-Nippon NVision-K5001). Three repeated measurements were performed with CST (Oculus Optikgeräte GmbH, Wetzlar, Germany, serial number 1.6b2507) in both eyes, using four different intervals between successive IOP and bIOP readings in the following order: 5 min (T5), 2 min (T2), 30 s (T30), and immediate succession (Ti). The first measurement in the T5 was called T5-1. T5-2 was performed 5 min after T5-1, and T5-3 was measured at 5 min after T5-2. The three measurements constituted one set of triplicate measurements. The same approach was applied to the T2 interval, with a 2-min interval between T2-1 and T2-2 and between T2-2 and T2-3. A 5-min resting period was provided between successive sets of triplicate measurements. All measurements for each participant were completed within a single session of approximately 30–40 min, and participants rested for at least 20 min before the first measurement. Given that the four protocols imposed strict time windows between successive measurements (5-min, 2-min, 30-s, and immediately, respectively), measurements had to be acquired within the designated interval to be valid. If eyes with frequent blinking or unstable fixation, the measurement occasionally could not be completed within the required window, resulting in incomplete data for that protocol. For each participant, the eye with complete measurements across all four protocols was designated as the study eye. When both eyes yielded complete datasets, the right eye was selected for analysis.

Data collection and eye examinations were carried out at the Tianjin Eye Hospital Optometric Centre. Written informed consents were obtained from the participants before data collection. The study was reviewed and approved by the Human Subjects Ethics Subcommittee of Nankai University (NKUIRB2024108), and all procedures were in agreement with the tenets of the Declaration of Helsinki.

### Sample size

The sample size was determined *a priori* using G*Power (version 3.1). Based on a repeated-measures ANOVA (*F* tests, within factors), a minimum of 76 participants was required to detect a small-to-moderate within-subject effect (effect size *f* = 0.15) across the four measurement protocols at a significance level of 0.05 with 80% power, assuming a correlation of 0.5 among repeated measures and a nonsphericity correction of *ε* = 0.75.

### Data analysis

Data analysis was conducted using SPSS (software version 27; SPSS Inc., Chicago, IL). Data normality was assessed with the Kolmogorov–Smirnov test. Normally distributed data was presented as the mean ± SD. Among 4 sets of triplicate IOP and bIOP measurements, some were not normally distributed even after Log and square root transformation, these are presented as median (interquartile range, IQR). A generalized linear mixed models (GLMM) was used to test the effect of measurement intervals (i.e., T5, T2, T30 and Ti) and three successive measurements at each interval. Whenever a significant effect was found, Friedman test was used to examine trends in IOP change across the three measurements within each interval. To evaluate the influence of baseline characteristics on IOP and bIOP, the GLMM was re-run with age, flat (K1) and steep (K2) corneal curvature, and SER or axial length (AL) as covariates, with SER and AL entered in separate models to avoid multicollinearity. Interaction terms between each statistically significant covariate and the two main effects (measurement intervals and successive measurement) were also examined. A *p* < 0.05 was considered statistically significant.

## Results

A total of 88 myopic participants were included in the analysis. The mean age was 21.5 ± 2.5 years (range: 19–31 years). A total of 68 right eyes and 20 left eyes were analysed. The mean SER was −4.49 ± 2.39 D (range: from −9.88 to −0.50 D). Baseline IOP was 16.0 (IQR: 2.9) mmHg, and bIOP was 15.9 (IQR: 2.5) mmHg.

**Figure 1:**
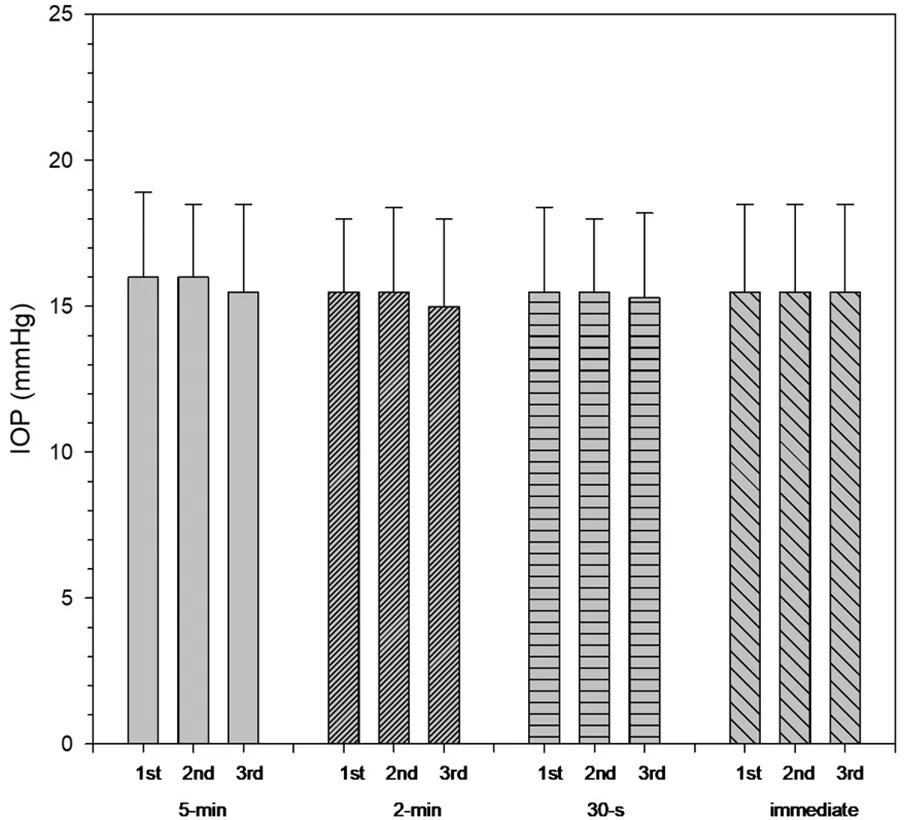
Intraocular pressure (IOP) at 5-min, 2-min- 30-s, and immediate after one another triplicate measurement intervals. The bars indicate median results and the error bars show interquartile ranges.

**Figure 2:**
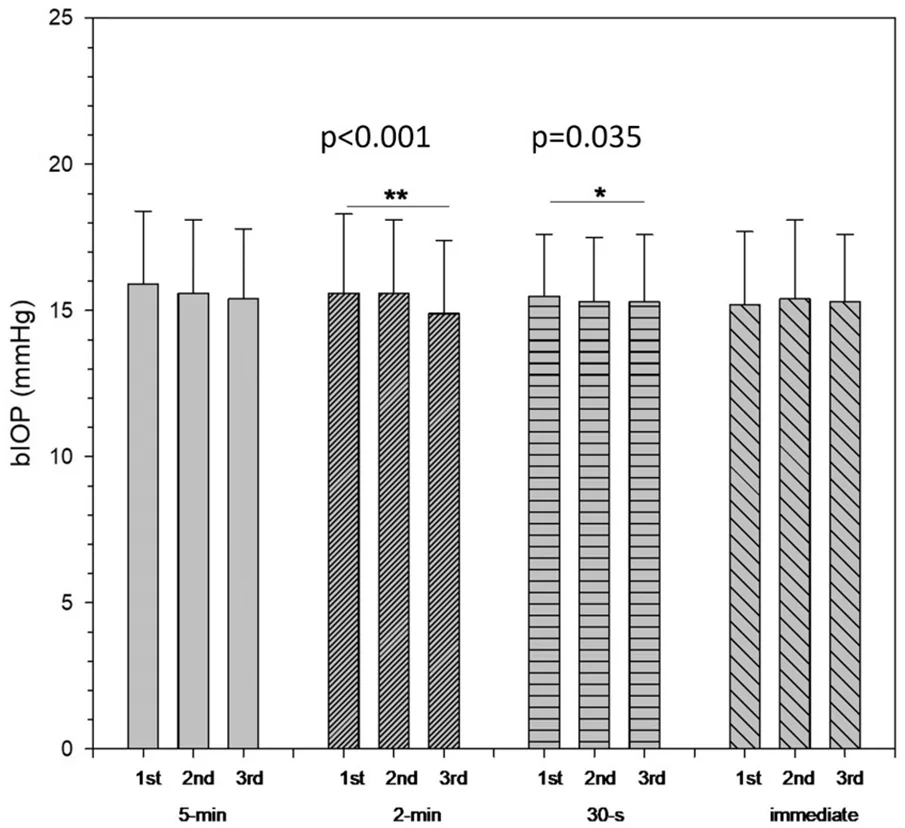
Biomechanically corrected intraocular pressure (bIOP) at 5-min, 2-min- 30-s, and immediate after one another triplicate measurement intervals. The bars indicate median results and the error bars show interquartile ranges. *p < 0.05, **p < 0.01.

Table 1 summariszes the baseline characteristics of all participants and Table 2 shows IOP and bIOP results. For IOP, GLMM revealed no significant order effect (*p* = 0.404) nor interval effect (*p* = 0.074, Table 3). Figure 1 shows the IOP results of the four sets of triplicate measurements. For bIOP, there was no significant successive measurement effect (*p* = 0.325). The effect of measurement interval was not reach to statistically significant level (*p* = 0.054, Table 4). Figure 2 shows the bIOP results of the four sets of triplicate measurements. Both 5-min and 2-min intervals showed a decreasing trend but only the 2-min interval reached statistical significant (Friedman test, *p* < 0.001). Pairwise comparisons revealed significant difference between T2-3 and T2-2 (*p* < 0.001), and between T2-3 and T2-1 (*p* < 0.001). Friedman test revealed a significant difference in the 30-s interval (*p* = 0.035) but pairwise comparisons did not identify any significantly different pairs.

**Table 1:** Mean or median of baseline characteristics of all participants.

Characteristic	Value
Participants, *n*	88
Age, years, mean ± SD (range)	21.5 ± 2.5 (19–31)
Eye, right/left, *n*	68/20
SER, D, mean ± SD (range)	−4.49 ± 2.39 (−9.88 to −0.50)
CCT, μm, mean ± SD (range)	554.0 ± 32.2 (480–632)
Flat keratometry (K1), D, mean ± SD (range)	42.90 ± 1.27 (39.63–46.14)
Steep keratometry (K2), D, mean ± SD (range)	44.23 ± 1.44 (41.19–47.82)
Axial length, mm, mean ± SD (range)	25.36 ± 1.15 (23.12–28.06)
Baseline IOP (T5-1), mmHg, median (IQR)	16.0 (2.9)
Baseline bIOP (T5-1), mmHg, median (IQR)	15.9 (2.5)

**Table 2:** Intraocular pressure (IOP) and biomechanically corrected IOP (bIOP) results at 5-min (T5), 2-min (T2), 30-s (T30), and immediate after one another (Ti).

Measurement	IOP (mmHg)		bIOP (mmHg)	
	Median	IQR	Median	IQR
T5-1	16.0	2.9	15.9	2.5
T5-2	16.0	2.5	15.6	2.5
T5-3	15.5	3.0	15.4	2.4
T2-1	15.5	2.5	15.6	2.7
T2-2	15.5	2.9	15.6	2.5
T2-3	15.0	3.0	14.9	2.5
T30-1	15.5	2.9	15.5	2.1
T30-2	15.5	2.5	15.3	2.2
T30-3	15.3	2.9	15.3	2.3
Ti-1	15.5	2.5	15.2	2.5
Ti-2	15.5	3.0	15.4	2.7
Ti-3	15.5	3.0	15.3	2.3

IQR, interquartile range.

**Table 3:** Generalized linear mixed model results for IOP.

Type III tests of fixed effects				
Source	Num. *df*	Den. *df*	*F*	*p*
Intercept	1	1,050	41,414.852	<0.001
Successive measurement	2	1,050	2.607	0.074
Measurement intervals	3	1,050	0.974	0.404

Estimates of fixed effects					
Parameter	Estimate	SE	*t*	*p*	95% CI
Intercept	15.79	0.19	82.468	<0.001	15.41–16.16
1st vs. 3rd successive measurement	0.44	0.19	2.278	0.023	0.06–0.81
2nd vs. 3rd consecutive measurement	0.24	0.19	1.276	0.202	−0.13–0.62
T5 vs. Ti	0.04	0.22	0.197	0.844	−0.39–0.48
T2 vs. Ti	−0.21	0.22	−0.960	0.338	−0.65–0.22
T30 vs. Ti	−0.27	0.22	−1.217	0.224	−0.70–0.16

**Table 4:** Generalized linear mixed model results for bIOP.

Type III tests of fixed effects				
Source	Num. *df*	Den. *df*	*F*	*p*
Intercept	1	1,050	61,099.752	<0.001
Successive measurement	2	1,050	2.926	0.054
Measurement intervals	3	1,050	1.157	0.325

Estimates of fixed effects					
Parameter	Estimate	SE	*t*	*p*	95% CI
Intercept	15.58	0.16	100.309	<0.001	15.27–15.88
1st vs. 3rd successive measurement	0.38	0.16	2.419	0.016	0.07–0.68
2nd vs. 3rd successive measurement	0.20	0.16	1.259	0.208	−0.11–0.50
T5 vs. Ti	0.04	0.18	0.211	0.833	−0.31–0.39
T2 vs. Ti	−0.18	0.18	−1.020	0.308	−0.53–0.17
T30 vs. Ti	−0.24	0.18	−1.346	0.179	−0.59–0.11

The influence of baseline characteristics on IOP and bIOP is summarized in Supplementary Tables S1–S4. For IOP, steep corneal curvature (K2: *F* = 32.362, *p* < 0.001), flat corneal curvature (K1: *F* = 6.668, *p* = 0.010), and SER (*F* = 14.830, *p* < 0.001) were significantly associated with IOP level, whereas age (*p* = 0.058) and AL (*p* = 0.061) were not. For bIOP, K2 (*F* = 14.097, *p* < 0.001) and SER (*F* = 5.754, *p* = 0.017) remained significantly associated, while K1, age, and AL showed no significant association (all *p* > 0.05). Importantly, after adjustment for these covariates, neither the interval effect nor the order effect showed any significant effect, and none of the interaction terms between the covariates and interval or order effects reached statistical significance (all *p* > 0.05).

## Discussion

The present study investigated the influence of successive measurement and measurement interval, on IOP and bIOP obtained with the CST in a cohort of young myopic adults. The study cohort comprised 68 right eyes and 20 left eyes. Given the high interocular symmetry of IOP and corneal biomechanical properties in healthy myopic individuals (30), this distribution is unlikely to have introduced systematic bias. IOP measured with CST was a stable parameter without any significant variation. Although a decreasing trend was observed in bIOP, the difference was less than 1 mmHg which is considered as clinically insignificant, since the within-subject standard deviation of CST has been reported as 0.98 mmHg for IOP and 0.89 mmHg for bIOP (31). Moreover, although corneal curvature and refractive status were associated with the absolute pressure level (32), they did not modify the stability of IOP and bIOP across repeated measurements.

Various studies have confirmed that repeated applanation tonometry leads to a reduction in IOP. This effect was first observed in the 1950s (10) and was summarised as “tonographic effect,” or “Bechrakis effect”. In subsequent decades, numerous studies using the GAT (13), Ocular Response Analyzer (8), have shown a similar phenomenon. Johannesson et al. (13) reported IOP reductions of 3.8 mmHg using GAT measurement and 4.4 mmHg using Applanation Resonance Tonometry (measurement in mean age of 47 and non-myopic adults). Zimmermann et al. (8) reported a mean IOP decrease of −0.55 ± 2.00 mmHg (*p* = 0.138) in glaucoma patients and −1.15 ± 1.52 mmHg (*p* < 0.001) for young healthy controls between the first and second GAT measurements. However, the interval between each measurement was not specified in either studies (8, 13). From our knowledge, this is the first study to investigate the effect on successive IOP and bIOP measurements using CST.

The CST device employs an ultra-high-speed Scheimpflug camera to capture the corneal response to a rapid air-puff impulse (33). Most previous studies examining repeated CST measurements have reported good repeatability and have typically averaged the results of repeated measurements without exploring the influence of the interval duration between measurements, despite intervals ranging from immediate succession up to 5 min (15, 16, 18, 19, 34). This oversight necessitates further investigation into how varying rest intervals impact measurement consistency and reliability. A reduction in IOP was reported in the Borroni et al. study (23), with a 21% reduction (13.75 mmHg to 10.84 mmHg) in 4 min and a 16% reduction (13.28 mmHg–11.11 mmHg) in 8 min after CST measurements using rebound tonometry in healthy and glaucoma middle-aged adults, respectively. Our cohort of young myopes exhibited a smaller bIOP reduction (approximately 0.5 mmHg at T2) compared with the Borroni study (approximately 2.91 mmHg in IOP) (23). One possible explanation is the use of rebound tonometry in Borroni study which may exhibit overestimation for high myopes (24, 25) or underestimation for low myopes (25). This discrepancy is attributed to the influence of altered corneal biomechanics on rebound tonometry, rather than solely to changes in CCT (26). The CST provides multiple dynamic corneal response parameters reflecting corneal biomechanical properties (35), and transient biomechanical changes induced by repeated air puffs could theoretically influence subsequent IOP measurements. Therefore, bIOP measured after different resting intervals was adopted as the primary outcome of our current study, as it minimizes the influence of corneal properties and potentially provides a more accurate assessment of IOP (27–29).

Similar to the IOP reduction achieved through repeated tonometry, ocular massage is a clinical technique in which gentle and controlled pressure is applied to the eyelid using a finger or a specialized device to lower IOP or to enhance the outflow of aqueous humour (36–38). Wu et al. (38) suggested that digital ocular massage applied sustained orbital pressure, and observed the reductions of IOP associated with Schlemm's canal expansion in adults with a median age of 20 years. A 42% decrease in IOP immediately after 10 s of digital ocular massage was reported in an older cohort (mean age: 59.5 years) (39). The impulse from CST causes substantial deformation of the cornea and anterior chamber, unlike the relatively milder applanation in GAT (33). Since the aqueous humour drains from the anterior chamber through the trabecular meshwork (TM), this deformation might alter the configuration of the anterior chamber, thereby temporarily increasing the outflow through the TM. It has been documented that air puff from CST exerted a maximum pressure of 95 mmHg at the corneal apex, diminishing radially over the central 8.0 mm of the cornea and lasting 22 ms (31, 40, 41). Therefore, the CST might be expected to cause a marked reduction in IOP. However, we observed no significant reduction in either IOP or bIOP. One possible explanation is that the eyeball rotated nasally and retracted during CST measurements, dissipating 30% of the applied force and reducing the TM stimulation (42), and that the impulse duration is much shorter than that of ocular massage. Hence, we hypothesized that CST may act as a weaker form of “ocular massage”, producing an IOP or bIOP reduction too small and transient to be clinically meaningful.

Apparently, both bIOP and IOP were quite stable among all measurements. These results are consistent with a previous study that used a 1-min interval, in which participants with a mean age of around 50 years showed no significant differences in IOP among three repeated measurements (13.9 ± 3.0 mmHg, 13.9 ± 3.0 mmHg, and 13.8 ± 3.4 mmHg) (22). A previous study suggested that after GAT measurement, anterior chamber volume returned to baseline within approximately 2 min, despite the observed failure of IOP to return to baseline levels even after 20 min of rest (13). Aqueous humour drainage from the anterior chamber through the TM into Schlemm's canal requires a finite duration to occur. We assumed that during corneal deformation induced by the air puff from the CST, aqueous humour is transiently displaced towards the TM. Subsequently, as the cornea rapidly rebounds to its original configuration, the anterior chamber volume is promptly restored. This rapid volume restoration may generate a transient negative pressure within the anterior chamber, which could contribute to the observed small bIOP reduction. However, as aqueous outflow through the TM and Schlemm's canal is not instantaneous, it regulates anterior chamber volume and maintains IOP homeostasis (43).

The limitations of the current study should be acknowledged. First, our participants were young and healthy myopes, which limits the findings cannot be directly generalized to patients with glaucoma or ocular hypertension, or to older populations, in whom corneal biomechanics and aqueous humour dynamics differ. Corneal biomechanical properties stiffen with age, and aqueous humour dynamics and trabecular meshwork outflow facility may be altered in older individuals and in patients with glaucoma (29). Future studies should include different age individuals as well as patients with glaucoma, with appropriate subgroup analyses and comparisons among different populations, to verify whether our findings can be extrapolated. In addition, the four measurement protocols were applied in a fixed order (T5, T2, T30, Ti) rather than in a randomized sequence, therefore, a carry-over effect from earlier protocols on subsequent measurements cannot be entirely excluded. The GLMM, which included interval and order as a factor, revealed no significant order effect on either IOP (*p* = 0.404) or bIOP (*p* = 0.325), and the small reduction in bIOP did not progress with the interval sequence, the largest decrease occurred at T2, whereas no significant change was observed in Ti, arguing against a cumulative effect of repeated air puffs. Nevertheless, the interval effect and the order effect cannot be fully dissociated in the present design, and future studies should randomize the order of measurement intervals to confirm our findings. Given that most Corvis ST parameters are themselves influenced by CCT, corneal curvature, and IOP (44), a comprehensive analysis of the full set of biomechanical parameters across four intervals and triplicate measurements constitutes a distinct research question that would substantially expand the scope of the present manuscript, which was specifically designed to address IOP behaviour; we therefore respectfully suggest that this question merits a dedicated study.

## Conclusions

Both IOP and bIOP measured with CST were stable across successive measurements. Although bIOP was decreased slightly with 2-min and 30-s intervals, the difference was small and not clinically significant.

## Data Availability

The raw data supporting the conclusions of this article will be made available by the authors, without undue reservation.
