# Supplementary material for: Investigating intraocular pressure fluctuations using corneal visualization Scheimpflug technology tonometry (CST) with varied resting intervals
Source: Front Med (Lausanne). 2026 Sep 9;13:1906060. doi: 10.3389/fmed.2026.1906060 (PMC13597312; doi:10.3389/fmed.2026.1906060)
Supplement: Supplementary file 1 [file Table1.docx]

**Supplementary Material**

Tables S1-S4: Type III tests of fixed effects from the generalized linear mixed models (GLMM) with baseline characteristics as covariates.

Successive measurement = the three successive measurements within each interval; measurement interval = the four resting-interval (T5, T2, T30, Ti). SER and AL were entered in separate models to avoid multicollinearity.

**Table S1. Fixed effects on IOP with age, corneal curvature, and SER as covariates**

| **Source** | **Num. df** | **Den. df** | **F** | **p** |
| --- | --- | --- | --- | --- |
| Intercept | 1 | 1031 | 2.349 | 0.126 |
| Successive measurement | 2 | 1031 | 0.321 | 0.725 |
| Measurement interval | 3 | 1031 | 0.403 | 0.751 |
| Age | 1 | 1031 | 3.594 | 0.058 |
| SER | 1 | 1031 | 14.830 | < 0.001 |
| K1 | 1 | 1031 | 6.668 | 0.010 |
| K2 | 1 | 1031 | 32.362 | < 0.001 |
| Successive measurement × SER | 2 | 1031 | 0.528 | 0.590 |
| Measurement interval × SER | 3 | 1031 | 0.228 | 0.877 |
| Successive measurement × K2 | 2 | 1031 | 0.068 | 0.934 |
| Measurement interval × K2 | 3 | 1031 | 0.810 | 0.488 |
| Successive measurement × K1 | 2 | 1031 | 0.113 | 0.893 |
| Measurement interval × K1 | 3 | 1031 | 0.550 | 0.648 |

**Table S2. Fixed effects on bIOP with age, corneal curvature, and SER as covariates**

| **Source** | **Num. df** | **Den. df** | **F** | **p** |
| --- | --- | --- | --- | --- |
| Intercept | 1 | 1036 | 5.962 | 0.015 |
| Successive measurement | 2 | 1036 | 0.276 | 0.759 |
| Measurement interval | 3 | 1036 | 0.733 | 0.532 |
| Age | 1 | 1036 | 0.294 | 0.588 |
| SER | 1 | 1036 | 5.754 | 0.017 |
| K1 | 1 | 1036 | 0.796 | 0.372 |
| K2 | 1 | 1036 | 14.097 | < 0.001 |
| Successive measurement × SER | 2 | 1036 | 0.603 | 0.547 |
| Measurement interval × SER | 3 | 1036 | 0.171 | 0.916 |
| Successive measurement × K2 | 2 | 1036 | 0.237 | 0.789 |
| Measurement interval × K2 | 3 | 1036 | 0.708 | 0.548 |

**Table S3. Fixed effects on IOP with age, corneal curvature, and axial length as covariates**

| **Source** | **Num. df** | **Den. df** | **F** | **p** |
| --- | --- | --- | --- | --- |
| Intercept | 1 | 1036 | 5.617 | 0.018 |
| Successive measurement | 2 | 1036 | 0.304 | 0.738 |
| Measurement interval | 3 | 1036 | 0.386 | 0.763 |
| Age | 1 | 1036 | 3.791 | 0.052 |
| AL | 1 | 1036 | 3.520 | 0.061 |
| K1 | 1 | 1036 | 6.665 | 0.010 |
| K2 | 1 | 1036 | 28.082 | < 0.001 |
| Successive measurement × K1 | 2 | 1036 | 0.062 | 0.940 |
| Measurement interval × K1 | 3 | 1036 | 0.495 | 0.686 |
| Successive measurement × K2 | 2 | 1036 | 0.026 | 0.975 |
| Measurement interval × K2 | 3 | 1036 | 0.781 | 0.505 |

**Table S4. Fixed effects on bIOP with age, corneal curvature, and axial length as covariates**

| **Source** | **Num. df** | **Den. df** | **F** | **p** |
| --- | --- | --- | --- | --- |
| Intercept | 1 | 1041 | 5.210 | 0.023 |
| Successive measurement | 2 | 1041 | 0.284 | 0.753 |
| Measurement interval | 3 | 1041 | 0.734 | 0.532 |
| Age | 1 | 1041 | 0.365 | 0.546 |
| AL | 1 | 1041 | 0.957 | 0.328 |
| K1 | 1 | 1041 | 0.755 | 0.385 |
| K2 | 1 | 1041 | 12.236 | < 0.001 |
| Successive measurement × K2 | 2 | 1041 | 0.261 | 0.770 |
| Measurement interval × K2 | 3 | 1041 | 0.720 | 0.540 |
